# Real-World Data on the Effectiveness and Safety of Filgotinib for Ulcerative Colitis in Japanese Patients: A Single-Center Experience

**DOI:** 10.7759/cureus.61496

**Published:** 2024-06-01

**Authors:** Takahito Toba, Ryo Karashima, Kodai Fujii, Keiichi Inoue, Nanako Inoue, Yurie Ogawa, Aya Hojo, Ai Fujimoto, Takahisa Matsuda

**Affiliations:** 1 Division of Gastroenterology and Hepatology, Toho University Omori Medical Center, Tokyo, JPN

**Keywords:** small-molecule agents, patient-reported outcome, janus kinase inhibitor, filgotinib, ulcerative colitis

## Abstract

Introduction

Filgotinib is a JAK-1 selective inhibitor approved for ulcerative colitis (UC) treatment in Japan. Its effectiveness has been confirmed but remains unknown in actual clinical practice. Therefore, we aimed to evaluate the effectiveness and safety of filgotinib and identify suitable patients in the Japanese population.

Methods

We retrospectively reviewed the background, clinical course, and laboratory data of patients treated with filgotinib 200 mg for UC between May 2022 and December 2023.

Results

The median observation period for the 25 patients was 232 days (interquartile ranges (IQR) 102-405). The median age of the patients was 43 years (IQR 29-55), disease duration was nine years (IQR 2-12), and 36% (9/25) of patients were biologic or small molecule naïve. The median patient-reported outcome (PRO2) and partial Mayo (pMayo) scores at agent initiation were 3 (IQR 1-4) and 4.5 (IQR 3-6), respectively. The PRO2 and pMayo scores improved significantly two weeks after treatment initiation (p < 0.05). Clinical remission rates at 24 weeks after treatment initiation were 60% (15/25) for PRO2 ≤ 1 and 52% (13/25) for pMayo ≤ 1. The Mayo endoscopic subscore significantly improved after filgotinib initiation (p=0.04), and the endoscopic remission rate was 47% (8/17). At 24 weeks, patients in clinical remission, compared to those not in remission, had significantly lower baseline PRO2 and pMayo scores and longer disease duration (p=0.03, p=0.03, and p=0.04, respectively). The filgotinib persistence rate was 68% (17/25), with no discontinuation because of adverse events. Patients who continued treatment had significantly lower PRO2, pMayo scores, and blood neutrophil counts at initiation than those who discontinued (p=0.02, p=0.03, and p=0.02, respectively).

Conclusion

Filgotinib appears to be effective and safe in Japanese patients with UC. Effectiveness and persistence were high in patients whose PRO2 and pMayo scores were low at the time of treatment initiation.

## Introduction

Ulcerative colitis (UC) is a chronic inflammatory bowel disease (IBD) with a remitting and relapsing course [1]. Recently, numerous biological and small-molecule agents for UC have emerged, and their effectiveness and safety have been demonstrated [2,3]. Filgotinib is a small-molecule Janus kinase (JAK) 1 preferential inhibitor approved for the treatment of moderately to severely active UC in Japan in March 2022. The international phase 2b/3 SELECTION trial (ClinicalTrials. gov ID: NCT02914522) evaluated the effectiveness and safety of filgotinib in comparison with a placebo in patients with moderately to severely active UC [4]. ​​​Additionally, several systematic reviews and network analyses have reported the effectiveness and safety of filgotinib [5-10]. However, there are few reports on real-world data in actual clinical practice. In the Japanese guidelines for IBD treatment [11], biologics and small-molecule agents are used in parallel for the treatment of refractory cases; however, it is not clear which agent is appropriate for which patient. Therefore, it is important to extract agent characteristics from real-world data. Consequently, we aimed to investigate the effectiveness and safety of filgotinib in Japanese patients with UC and to identify patients suitable for filgotinib in real-world clinical practice.

## Materials and methods

This was a single-center, retrospective, observational cohort study. We investigated the background, clinical course, and laboratory data of all 25 patients treated with filgotinib 200 mg for UC at our hospital (Toho University Omori Medical Center, Tokyo) between May 2022 and December 2023. Patient data were collected from the electronic medical records of the hospital.

Definitions

Biologics (BIO)/JAK multi-failure was defined as the primary or secondary ineffectiveness of three or more agents. Agents with the same mechanism, such as infliximab and adalimumab, which are anti-tumor necrosis factor-alpha (TNF-α) antibodies, were counted as different agents. Clinical remission was defined as achieving a patient-reported outcome (PRO2) score or a partial Mayo (pMayo) score of ≤ 1 and a rectal bleeding subscore of 0. Endoscopic remission was defined as an endoscopic subscore of 0 or 1. Serious adverse events were defined as any event leading to permanent discontinuation of therapy, hospitalization, or death. Moderate adverse events were defined as any event requiring temporary discontinuation or dose reduction. All other adverse events were defined as mild.

Assessment

We examined clinical remission rates at 2, 8, and 24 weeks after the initiation of filgotinib, PRO2, and pMayo scores over time after the initiation of filgotinib, change in the Mayo endoscopic subscore (MES) after filgotinib initiation, endoscopic remission rate, and the safety and persistence of filgotinib. Changes in the MES and endoscopic remission rate were examined in 17 patients who performed endoscopic evaluation before filgotinib initiation. To evaluate clinical remission and persistence, the background factors in remission and non-remission groups and the persistent and non-persistent groups were also analyzed.

Statistical analysis

Statistical analyses were performed using R software (http://www. r-project. org). Continuous variables are expressed as medians with interquartile ranges (IQR), and categorical variables are expressed as frequencies with percentages. Continuous and categorical variables were compared using the Wilcoxon rank-sum test and chi-squared test, respectively. Clinical remission rates were evaluated by intention-to-treat analysis, the change in the MES after filgotinib initiation was evaluated using a paired t-test, and filgotinib persistence was assessed using Kaplan-Meier analysis; patients were censored at the last follow-up. Statistical significance was set at p < 0.05.

## Results

Clinical characteristics of the patients

Clinical characteristics of the 25 patients at the time of filgotinib initiation are presented in Table 1. The median age was 43 years (IQR 29-55) and 64% (16/25) of the patients were male. The median disease duration was nine years (IQR 2-12), and the most common type of disease was the extensive type 76% (19/25), with no cases of proctitis. Treatment histories included 36% (9/25) biologic/JAK-naïve cases, 52% (13/25) multi-failure cases, 32% (8/25) steroid-dependent cases, and 36% (9/25) steroid-resistant cases. 5-aminosalicylate (5-ASA) was the most common concomitant drug (64% 16/25), with concomitant thiopurine in 20% (5/25) of the patients. The median PRO2 score was 3 (IQR 1-4), the median pMayo score was 4.5 (IQR 2.75-6), the serum C-reactive protein (CRP) level was 0.2 mg/dL (IQR 0.1-0.525), and the albumin level was 4.2 (IQR 3.7-4.325) at the time of filgotinib initiation. Endoscopy was performed before induction in 68% (17/25) of the patients with a median MES of 2 (IQR 2-3).

**Table 1:** Patient characteristics at the time of filgotinib initiation. 5-ASA: 5-aminosalicylates, BIO: biologics, JAK: janus kinase, PRO2: patient-reported outcomes, MES: Mayo endoscopic subscore, CRP: C-reactive protein, IQR: interquartile range, WBC: white blood cell, Hgb: hemoglobin, Plt: platelet. *Missing data: 1, **Missing data: 8 The data has been represented as N(%), median(IQR).

N=25	
Age, years, median (IQR)	43 (29-55)
Male, n (%)	16 (64)
Disease duration, years, median (IQR)	9 (2-12)
Disease extent, n (%)	
- Extensive	19 (76)
- Left-sided	6 (24)
- Proctitis	0 (0)
Smoker, n (%)	5 (20)
Drinker, n (%)	9 (36)
Steroid dependent, n (%)	8 (32)
Steroid resistant, n (%)	9 (36)
5-ASA intolerant, n (%)	7 (28)
Thiopurine intolerant, n (%)	9 (36)
BIO/JAK naïve, n (%)	9 (36)
BIO/JAK multi-failure, n (%)	13 (52)
Concomitant 5-ASA, n (%)	16 (64)
Concomitant thiopurine, n (%)	5 (20)
Concomitant prednisolone, n (%)	5 (20)
PRO2 score, median (IQR) *	3 (1-4)
Partial Mayo score, median (IQR) *	4.5 (2.75-6)
MES, median (IQR) **	2 (2-3)
CRP mg/dL, median (IQR) *	0.2 (0.1-0.525)
Albumin g/dL, median (IQR) *	4.2 (3.7-4.325)
WBC /μL, median (IQR) *	6400 (5500-7700)
Neutrophils /μL, median (IQR) *	3795 (2832-5328)
Lymphocytes /μL, median (IQR) *	1936 (1316-2688)
Hgb g/dL/μL, median (IQR) *	13.7 (12.2-14.8)
Plt ×10^4^/μL, median (IQR) *	30.6 (26.7-35)

Effectiveness of filgotinib

**Figure 1:**
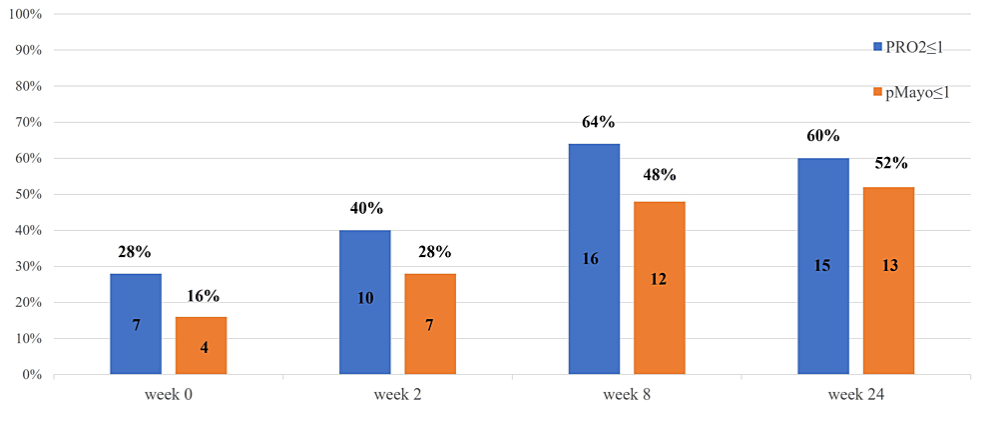
Clinical remission (PRO2 ≤ 1 or pMayo ≤ 1) at weeks 0, 2, 8, and 24 from filgotinib initiation (intention-to-treat analysis, N=25). PRO2: patient-reported outcomes, pMayo: partial Mayo. The data has been represented as N(%).

Changes in activity parameters over time at 2, 8, and 24 weeks after filgotinib initiation are presented in Table 2, and clinical remission rates are shown in Figure 1. The median PRO2 and pMayo scores improved significantly after two weeks of filgotinib initiation compared to that at week 0, and the scores improved further after 8 and 24 weeks (p < 0.05). CRP levels did not decrease significantly at weeks 2 and 8 but decreased significantly at 24 weeks compared to that at week 0 (p < 0.05).

**Table 2:** Changes over time in the PRO2 score, partial Mayo score, and CRP after filgotinib initiation. PRO2: patient-reported outcomes, CRP: C-reactive protein, IQR: interquartile range. *: p<0.05, Compared with week 0. The data has been represented as median (IQR). The p-value was considered significant at p < 0.05.

	Week 0	Week 2	Week 8	Week 24
PRO2 score, median (IQR)	3 (1-4)	1.5 (0-3)*	0 (0-1)*	0 (0-0)*
Partial Mayo score, median (IQR)	4.5 (3-6)	3 (0-5)*	1 (0-2)*	0 (0-1)*
CRP mg/dl, median (IQR)	0.2 (0.1-0.58)	0.2 (0-0.4)	0.1 (0-0.175)	0 (0-0.075)*

The clinical remission rates were 28% (7/25), 40% (10/25), 64% (16/25), and 60% (15/25) for weeks 0, 2, 8, and 24, respectively, for PRO2 scores and 16% (4/25), 28% (7/25), 48% (12/25), and 52% (13/25) for pMayo scores, respectively.

Comparison of factors correlated with clinical remission at 24 weeks between patients in remission and those not in remission showed significant differences in low PRO2 and pMayo score at initiation of filgotinib and long-term disease duration, respectively (p=0.03, p=0.03 and p=0.04). No other factors correlated with clinical remission (Table 3 and Table 4). Changes in the MES after filgotinib initiation are shown in Table 5. MES significantly improved after filgotinib initiation (p=0.04). The endoscopic remission rate was 47% (8/17).

**Table 3:** Comparison of clinical background of PRO2 remission and non-remission groups after 24 weeks of filgotinib initiation. 5-ASA: 5-aminosalicylates, BIO: biologics, JAK: janus kinase, PRO2: patient-reported outcomes, MES: Mayo endoscopic subscore, CRP: C-reactive protein, WBC: white blood cell, Hgb: hemoglobin, Plt: platelet, IQR: interquartile range, *: p<0.05. The data has been represented as N(%), median(IQR). The p-value was considered significant at p < 0.05.

	Remission (N=15)	Non-remission (N=10)	p
Age, years, median (IQR)	43 (32-55.5)	32.5 (24.5-48.3)	0.26
Male, n (%)	8 (53.3)	8 (80)	0.23
Disease duration, years, median (IQR)	11 (3.5-14.5)	5.5 (2.0-8.5)	0.04*
Disease extent, n (%)			0.65
- Extensive	12 (80)	7 (70)	
- Left-sided	3 (20)	3 (30)	
- Proctitis	0 (0)	0 (0)	
Smoker, n (%)	3 (20)	2 (20)	1
Drinker, n (%)	5 (33.3)	4 (40)	1
Steroid dependent, n (%)	5 (33.3)	3 (30)	1
Steroid resistant, n (%)	4 (26.7)	5 (50)	0.44
5-ASA intolerant, n (%)	5 (33.3)	2 (20)	0.79
Thiopurine intolerant, n (%)	5 (33.3)	4 (40)	1
BIO/JAK naïve, n (%)	5 (33.3)	4 (40)	1
BIO/JAK multi-failure, n (%)	8 (53.3)	5 (50)	1
Concomitant 5-ASA, n (%)	10 (66.7)	6 (60)	1
Concomitant thiopurine, n (%)	3 (20)	2 (20)	1
PRO2 score, median (IQR)	2 (0.5-3)	3.5 (3-4)	0.03*
MES, median (IQR)	2 (2-2.5)	2 (2-2.75)	0.68
CRP mg/dL, median (IQR)	0.2 (0.1-0.4)	0.2 (0.1-0.55)	1
Albumin g/dL, median (IQR)	4.2 (3.75-4.3)	4.1 (3.75-4.375)	0.98
WBC /μL, median (IQR)	6400 (6150-7600)	6700 (5275-9350)	0.89
Neutrophils /μL, median (IQR)	3795 (2960-4614)	4369 (2555-6209)	0.72
Lymphocytes /μL, median (IQR)	1976 (1550-2604)	1656 (1273-2785)	0.61
Hgb g/dL, median (IQR)	12.7 (11.9-14.3)	14.2 (13.8-14.8)	0.31
Plt ×10^4^/μL, median (IQR)	31.7 (29.1-35.0)	26.9 (24.5-33.4)	0.24

**Table 4:** Comparison of clinical background of partial Mayo remission and non-remission groups after 24 weeks of filgotinib initiation. 5-ASA: 5-aminosalicylates, BIO: biologics, JAK: janus kinase,  MES: Mayo endoscopic subscore, CRP: C-reactive protein, WBC: white blood cell, Hgb: hemoglobin, Plt: platelet, IQR: interquartile range, *: p<0.05. The data has been represented as N(%), median(IQR). The p-value was considered significant at p < 0.05

	Remission (N=13)	Non-remission (N=12)	p
Age, years, median (IQR)	43 (34-55)	32.5 (25.5-50.5)	0.31
Male, n (%)	6 (46.2)	10 (83.3)	0.1
Disease duration, years, median (IQR)	11 (4-13)	5.5 (2-9)	0.04*
Disease extent, n (%)			0.38
- Extensive	11 (84.6)	8 (66.7)	
- Left-sided	2 (15.4)	4 (33.3)	
- Proctitis	0	0	
Smoker, n (%)	2 (15.9)	3 (25)	0.65
Drinker, n (%)	4 (30.8)	5 (41.7)	1
Steroid dependent, n (%)	5 (38.5)	3 (25)	0.67
Steroid resistant, n (%)	3 (23.1)	6 (50)	0.23
5-ASA intolerant, n (%)	4 (30.8)	3 (25)	1
Thiopurine intolerant, n (%)	4 (30.8)	5 (41.7)	0.69
BIO/JAK naïve, n (%)	9 (69.2)	7 (58.3)	0.69
BIO/JAK multi-failure, n (%)	7 (53.8)	6 (50)	1
Concomitant 5-ASA, n (%)	9 (69.2)	7 (58.3)	0.69
Concomitant thiopurine, n (%)	2 (15.9)	3 (25)	0.65
Partial Mayo score, median (IQR)	3 (1-4)	5 (4.75-6)	0.03*
MES, median (IQR)	2 (2-2.75)	2 (2-2.5)	0.86
CRP mg/dL, median (IQR)	0.2 (0.1-0.5)	0.2 (0.1-0.45)	0.91
Albumin g/dL, median (IQR)	4.2 (3.8-4.3)	4.1 (3.7-4.325)	0.94
WBC /μL, median (IQR)	6400 (6000-6900)	7500 (5425-9250)	0.43
Neutrophils /μL, median (IQR)	3540 (2832-4466)	4602 (3105-6732)	0.35
Lymphocytes /μL, median (IQR)	1976 (1560-2520)	1656 (1243-3057)	0.69
Hgb g/dL, median (IQR)	12.7 (11.6-13.7)	14.2 (13.4-15.0)	0.18
Plt ×10^4^/μL, median (IQR)	30.9 (28.8-34.6)	27.8 (24.6-35.1)	0.54

**Table 5:** Change in the MES after filgotinib initiation (paired t-test, N=17). MES: Mayo endoscopic subscore, SD: Standard deviation The data has been represented as mean±SD. The p-value was considered significant at p < 0.05.

	Baseline	After treatment	p
MES, mean±SD	2.18±0.73	1.44±0.98	0.04

Persistence of filgotinib

**Figure 2:**
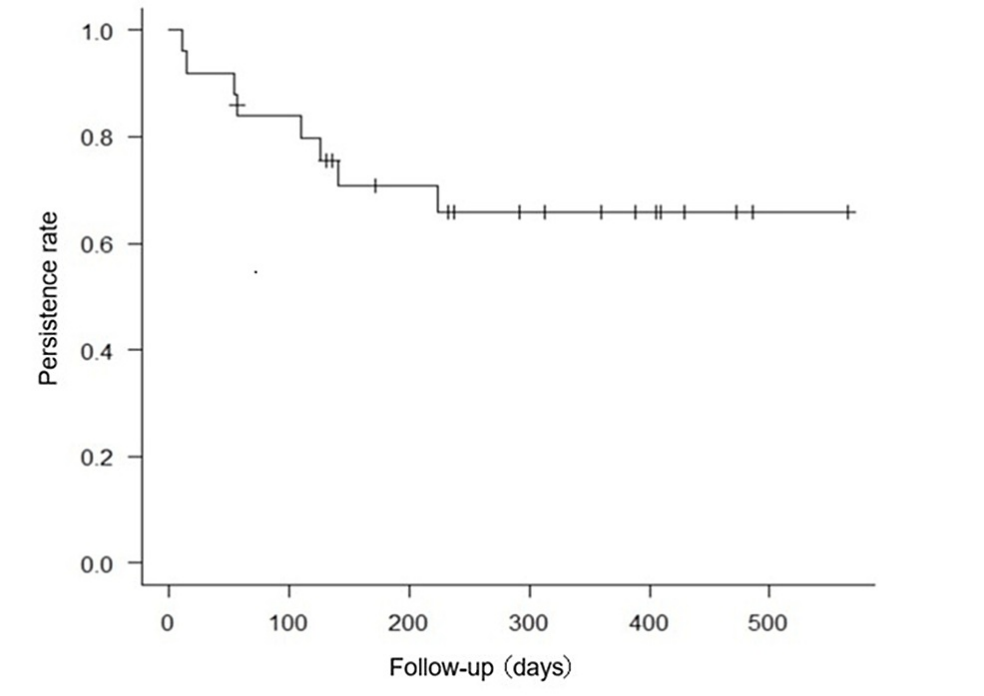
Kaplan–Meier curve for filgotinib persistence.

The median duration of observation was 232 days (IQR 126-405), and the filgotinib persistence rate at the end of the observation period was 68% (17/25). The median time to filgotinib discontinuation in the discontinued cases was 83 days (IQR 44-129.75) (Figure 2). The reasons for discontinuation were primary non-response in 87.5% (7/8) patients and secondary non-response in 12.5% (1/8) patients, with no discontinuation because of adverse events.

Comparative data for the persistence and discontinuation groups are presented in Table 6​​​​​​​. Significant patient characteristics related to persistence were long disease duration (p=0.04), low PRO2 (p=0.02), low pMayo scores (p=0.03), and low blood neutrophils (p=0.02) at initiation. There was also a trend toward greater discontinuation of steroid resistance (p=0.06).

**Table 6:** Comparison of patient characteristics in filgotinib persistence and discontinuance groups. 5-ASA: 5-aminosalicylates, BIO: biologics, JAK: janus kinase, PRO2: patient-reported outcomes, MES: Mayo endoscopic subscore, CRP: C-reactive protein, WBC: white blood cell, Hgb: hemoglobin, Plt: platelet, IQR: interquartile range, *: p<0.05, **: p<0.1. The data has been represented as N(%), median(IQR). The p-value was considered significant at p < 0.05, and the p-value < 0.1 was considered to indicate a trend.

	Persistence (N=17)	Discontinuance (N=8)	p
Age, years, median (IQR)	43 (30-55)	37.5 (25.5-50.5)	0.54
Male, n (%)	10 (58.8)	6 (75)	0.43
Disease duration, years, median (IQR)	11 (4-13)	4 (2-7.5)	0.04*
Disease extent, n (%)			0.36
- Extensive	12 (71)	7 (87.5)	
- Left-sided	5 (29)	1 (12.5)	
- Proctitis	0 (0)	0 (0)	
Smoker, n (%)	4 (24)	1 (12.5)	0.52
Drinker, n (%)	7 (41)	2 (25)	0.42
Steroid dependent, n (%)	6 (35)	2 (25)	0.6
Steroid resistant, n (%)	4 (24)	5 (62.5)	0.06**
5-ASA intolerant, n (%)	5 (29)	2 (25)	0.81
Thiopurine intolerant, n (%)	7 (41)	2 (25)	0.43
BIO/JAK naïve, n (%)	6 (35)	3 (37.5)	0.91
BIO/JAK multi-failure, n (%)	9 (53)	4 (50)	0.89
Concomitant 5-ASA, n (%)	12 (71)	4 (50)	0.31
Concomitant thiopurine, n (%)	3 (18)	2 (25)	0.67
Concomitant prednisolone, n (%)	3 (18)	2 (25)	0.67
PRO2 score, median (IQR)	2 (0.75-3.25)	3.5 (3-4)	0.02*
Partial Mayo score, median (IQR)	3 (1.75-5.25)	5.5 (5-6)	0.03*
MES, median (IQR)	2 (2-2.5)	2 (2-2.75)	0.67
CRP mg/dL, median (IQR)	0.2 (0.1-0.425)	0.25 (0.1-0.675)	0.68
Albumin g/dL, median (IQR)	4.2 (3.775-4.425)	4.1 (3.575-4.3)	0.44
WBC /μL, median (IQR)	6400 (5000-11500)	9200 (7050-9725)	0.03*
Neutrophils /μL, median (IQR)	3380 (2300-3968)	5918 (4849-7525)	0.02*
Lymphocytes /μL, median (IQR)	1936 (1440-2520)	1704 (1301-3057)	0.8
Hgb g/dL, median (IQR)	12.8 (12.2-14.3)	14.0 (12.6-14.975)	0.47
Plt ×10^4^/μL, median (IQR)	30.6 (26.7-34.2)	31.8 (26.2-35.65)	0.75

Safety of filgotinib

Adverse events are listed in Table 7. Moderate or minor adverse events were observed in four patients (16% 4/25). Moderate adverse events, such as Ramsay-Hunt syndrome, herpes zoster, and renal dysfunction, were observed in one patient each, requiring temporary discontinuation of filgotinib or dose reduction from 200 to 100 mg. Details of renal dysfunction were provided by a 0.2 mg/dL elevation in serum creatinine from baseline after filgotinib initiation. Dose reduction resulted in rapid improvement. Minor adverse events included nausea in one patient. All patients recovered, and no permanent discontinuations because of adverse events were required. No serious adverse events were reported.

**Table 7:** Adverse events. The data has been represented as N(%).

Events	N=25
Mild	1 (4%)
Nausea	1 (4%)
Moderate	3 (12%)
Herpes Zoster infection (including Ramsay-Hunt syndrome)	2 (8%)
Renal dysfunction	1 (4%)
Serious	0 (0%)
Hospitalization	0 (0%)
Death	0 (0%)

## Discussion

This is the first study to demonstrate the effectiveness and safety of filgotinib in Japanese patients with UC using data from single-center various real-world clinical situations. Our results complemented those of randomized controlled trials. Another novelty of this study is the addition of the PRO2 assessment of clinical activity to the pMayo score; the PRO2 score is evaluated using only two items, the rectal bleeding score and stool frequency score, reported by the patients themselves. It is considered more objective as it eliminates the subjective assessment of the healthcare provider, correlates with endoscopic healing, and is recommended in STRIDE II [12]. There are several reports on other agents for UC that use PRO2 assessment [13].

The clinical remission rates in this study were as high as for PRO2 remission 40% (10/25) at week 2, 64% (16/25) at week 8, 60% (15/25) at week 24, for pMayo remission 28% (7/25) at week 2, 48% (12/25) at week 8, and 52% (13/25) at week 24. The endoscopic remission rate was also high at 47% (8/17). Japanese data in the post hoc analysis of the SELECTION trial reported a clinical remission rate of 26.7% and an endoscopic remission rate of 13.3% at week 10 in BIO/JAK-naïve patients [14]. This discrepancy may be because of the relatively low clinical activity in patients at the time of filgotinib initiation in this study. In a post hoc analysis of Japanese data from the SELECTION trial, the average Total Mayo Clinic score at filgotinib initiation was 8-9 and a high proportion had an endoscopic subscore of 3 at baseline, indicating relatively high disease activity. In contrast, in the present study, the median pMayo score at initiation was 4.5 and the proportion of baseline MES3 was 29% (5/17). Additionally, the clinical remission rate and its sustainability were also significantly correlated with the relatively low clinical activity at initiation. Furthermore, there was a tendency for early treatment discontinuation owing to inadequate efficacy, particularly in severe cases. Thus, these results suggest that filgotinib may be a promising option for patients with moderate-to-severe UC who exhibit relatively mild disease activity.

This study included 36% (9/25) BIO/JAK-naïve and 52% (13/25) BIO/JAK multi-failure cases; however, these factors did not affect the efficacy or persistence of filgotinib, consistent with the results of the post hoc analysis of the SELECTION trial [15]. Various cytokines are involved in the pathogenesis of UC, and cytokine profiles differ depending on the disease severity [2,16]. In severe cases, TNF-α is often highly expressed, and filgotinib, the mildest JAK-1 selective inhibitor among JAK inhibitors [17], may be less effective in such cases. The same reason may explain the discontinuance cases with high blood neutrophils. In contrast, the Janus kinase-signal transducer and activator of transcription (JAK-STAT) pathway may be a major cascade of inflammation in cases with relatively mild activity and long disease duration, as in this study. Therefore, we considered filgotinib to be highly effective and persistent in a population with relatively mild activity regardless of previous treatment. The trend towards increased discontinuation in steroid-resistant cases also suggests that it is difficult to maintain long-term effectiveness by simply suppressing the JAK-STAT pathway in steroid-resistant cases.

A post hoc analysis of the SELECTION trial reported that the concomitant use of immunomodulators did not affect the efficacy or safety of filgotinib [18]. Similarly, in the present study, 5-ASA, thiopurine, and prednisolone did not affect the effectiveness or adverse effects of filgotinib. This may be because immunomodulators and prednisolone have overlapping mechanisms of action with filgotinib, or because filgotinib is not an immunogenic agent.

The incidence of adverse events in this study was 16% (4/25). All cases were mild-to-moderate, with no treatment interruption owing to adverse events, and the results were considered safe. Moreover, filgotinib is assumed to reduce adverse events by selectively inhibiting JAK-1. The SELECTION trial also reported no difference in the incidence of adverse events compared to that with placebo, consistent with the fact that the majority of patients had mild to moderate disease [4]. However, two of the four adverse events were herpes zoster. Asians have been cited as being at risk for herpes zoster with JAK inhibitors [19]. Therefore, caution is warranted with filgotinib use. Additionally, filgotinib has been associated with concerns regarding the occurrence of thrombosis, elevated cholesterol levels, and rhabdomyolysis [20]. However, these were not observed in this study. Moreover, reports on actual clinical data for filgotinib in Scotland [21] showed that the frequency and profile of adverse reactions were similar to those in the present study, supporting the report that there were no differences in filgotinib pharmacokinetics by race in a Phase I study [22].

This study demonstrates the effectiveness and safety of filgotinib in Japanese patients with UC, which could broaden the scope of future treatments for UC. However, this study had some limitations. This study was based on retrospective, single-center data, which may limit its general applicability. In addition, the limited sample size and short follow-up period made it challenging to address the long-term effectiveness and safety of filgotinib. The study also had several missing data values, with incomplete endoscopic mucosal assessment of treatment effectiveness, and the timing of endoscopic treatment effectiveness varied from 2 to 12 months after filgotinib initiation. Furthermore, changes in biomarkers such as fecal calprotectin and the assessment of histologic remission are not available. Therefore, future studies are required to address these limitations.

## Conclusions

In conclusion, this study demonstrated the favorable effectiveness, safety, and persistence of filgotinib in Japanese patients with UC in clinical practice. Our results suggest that filgotinib may be particularly suitable for patients with moderate-to-severe disease exhibiting relatively mild activity. Given the wide array of therapeutic options available for UC, determining the most appropriate medication for each patient is critically important. This study contributes to the growing body of evidence needed to guide clinicians in making informed treatment decisions, ensuring that patients receive the most effective and safe therapy tailored to their specific clinical profiles.
